# Fort Sherman Virus Infection in Human, Peru, 2020

**DOI:** 10.3201/eid3010.240124

**Published:** 2024-10

**Authors:** Edmilson F. de Oliveira-Filho, César Augusto Cabezas Sánchez, Dora Esther Valencia Manosalva, Maribel Dana Figueroa Romero, Nancy Susy Merino Sarmiento, Adolfo Ismael Marcelo Ñique, Edward Málaga-Trillo, Andres Moreira-Soto, Maria Paquita García Mendoza, Jan Felix Drexler

**Affiliations:** Charité-Universitätsmedizin Berlin, Freie Universität Berlin and Humboldt-Universität zu Berlin, Institute of Virology, Berlin, Germany (E.F. de Oliveira-Filho, A. Moreira-Soto, J.F. Drexler);; Instituto Nacional de Salud, Lima, Peru (C.A. Cabezas Sánchez, M.D. Figueroa Romero, N.S. Merino Sarmiento, A.I.M. Ñique, M.P. Garcia Mendoza);; Laboratorio de Referencia Regional de Salud Pública de Lambayeque, Lambayeque, Perú (D.E. Valencia Manosalva);; Universidad Peruana Cayetano Heredia, Lima (E. Málaga-Trillo);; German Centre for Infection Research, Berlin (J.F. Drexler)

**Keywords:** Fort Sherman virus, viruses, arbovirus, zoonoses, febrile disease, orthobunyavirus, Peru

## Abstract

Fort Sherman virus (FSV) was isolated in Panama in 1985 from a US soldier. We report a case of human FSV infection in a febrile patient from northern coastal Peru in 2020. FSV infections spanning ≈35 years and a distance of 2,000 km warrant diagnostics, genomic surveillance, and investigation of transmission cycles.

In 1985, the orthobunyavirus Fort Sherman virus (FSV) was discovered in a US soldier with acute febrile disease who was based in a jungle warfare training center in Panama (*1*). Two FSV strains were isolated from mosquitoes in Argentina in 1965 and 1982 (*2*). FSV was found in healthy horses in Brazil in 2018, (*2*) and in horses in Argentina showing neurologic and abortive disease in 2013 (*3*). Serologic analyses of horse-associated FSV strains have suggested a broad vertebrate host range in peridomestic animals; seroprevalence has ranged from 2.9% in goats to 22.0% in horses in Brazil (*2*) and 5.7% in humans in Argentina (*4*).

We describe a case of human FSV infection in a patient with febrile illness sampled in March 2020 in the city of Chiclayo in Lambayeque department on the northern coast of Peru (Figure 1). The patient was a 61-year-old man with no recent travel history and fever of 38°C. Results of diagnostic tests were negative, including dengue virus (DENV)–specific real-time reverse transcription PCR (RT-PCR) and broadly reactive nested RT-PCRs targeting flaviviruses and alphaviruses. Expanded diagnostic investigation yielded a positive result for orthobunyaviruses using a broadly reactive RT-PCR (Appendix). We identified the virus as FSV by sequencing of the screening PCR amplicon (Appendix). We obtained complete coding sequences of all 3 genome segments by amplifying overlapping genome fragments using nested RT-PCR, followed by Sanger sequencing (Appendix). Virus isolation failed despite repeated attempts, potentially because of sample degradation and a relatively low viral load of 3.7 × 10^2^ viral RNA copies/mL of blood quantified using published FSV-specific real-time RT-PCR (*2*). 

**Figure 1:**
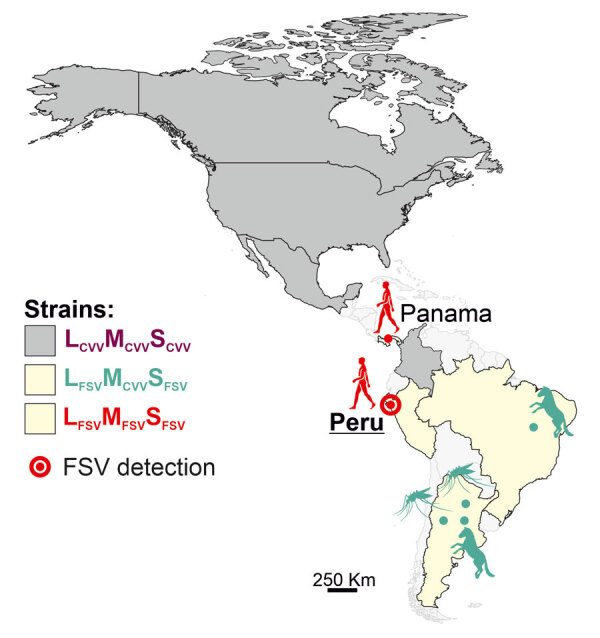
Geographic distribution of CVV and both FSV strains along the North and South American continents in study of FSV infection, Peru, 2020. Additional information on the sequences used to build the figure is provided (Appendix Table 1). CVV, Cache Valley virus; FSV, Fort Sherman virus; L, large segment; M, medium segment; S, small segment.

To investigate the extent of FSV infection in Lambayeque, we examined all 582 available serum samples from febrile persons sent for diagnostics to the local reference laboratory from Peru’s Ministry of Health during 2020 using RT-PCR for orthobunyaviruses. Of the samples, 70.4% (410/582 [95% CI 66.6–74.0]) tested positive for DENV, but no samples tested positive for FSV, other orthobunyaviruses, alphaviruses, or other flaviviruses (Appendix Table 3, Figure).

The genetic identity of the human-derived FSV strains from Panama and Peru was notable because those 2 strains were sampled over a distance of 2,000 km and nearly 4 decades apart. Nucleotide distances of the complete coding sequences compared with the prototypic FSV were 2.0% for large, 2.3% for medium (M), and 1.0% for small gene sequences. Translated amino acid sequence distances were low at all coding sequences, ranging from 0 to 1.5% (Appendix Table 4), which is compatible with strong purifying selection acting on arthropodborne viruses, such as FSV (*5*).

In phylogenetic reconstructions, the Peru FSV clustered with the Panama FSV prototype strain in all 3 viral genes. In the M gene–based phylogeny, the Panama and Peru FSV strains were monophyletic and nested in the Cache Valley virus (CVV) clade with robust bootstrap support (Figure 2). In contrast, mosquito- and horse-derived FSV strains from Argentina and Brazil differed from the FSV prototype in the phylogeny of their glycoprotein-encoding M gene (Figure 2). Phylogenetic inference of human-derived strains suggested an evolutionary origin of M genes involving a nonrecent reassortment event involving CVV (*2*). CVV frequently infect ruminants in North America, causing severe disease and congenital defects (*6*). Febrile disease in CVV-infected humans has been reported sporadically (*6*). The range of potential vertebrate or invertebrate hosts in which FSV and CVV reassortment might have occurred is thus wide.

**Figure 2:**
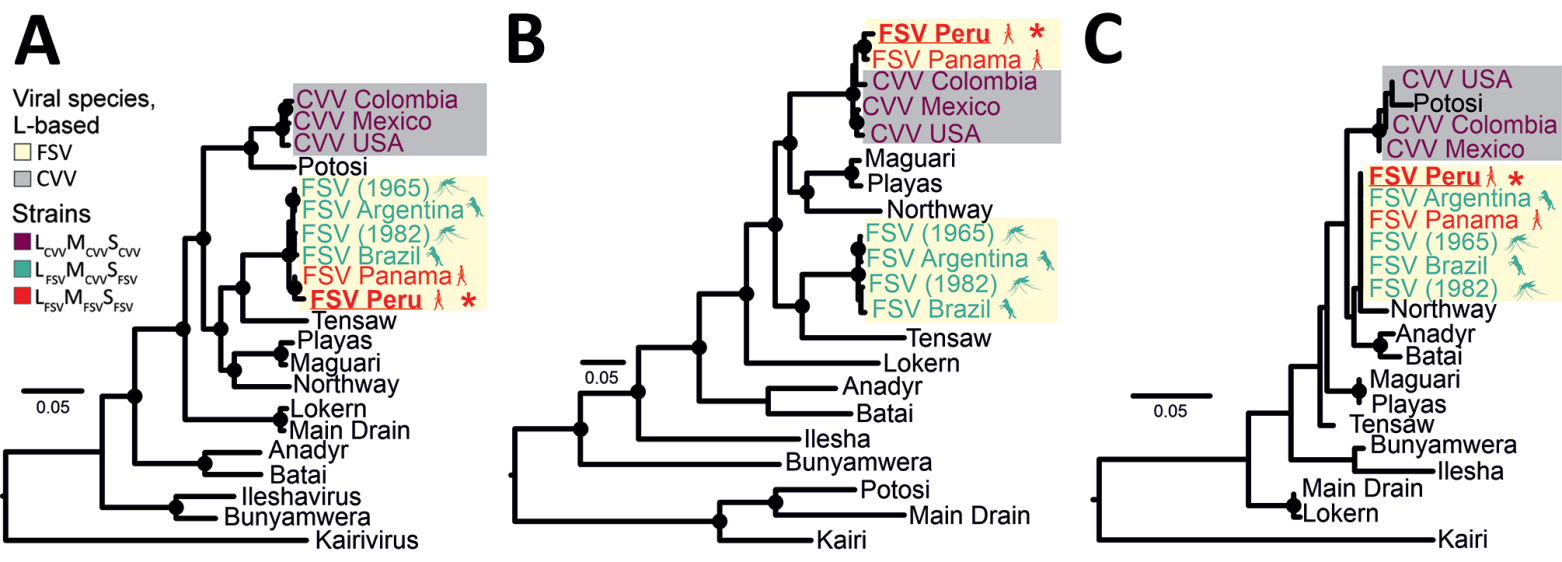
Maximum-likelihood phylogenetic trees based on deduced amino acid sequences of the L (A), M (B), and S (C) gene segments in study of Fort Sherman virus infection, Peru, 2020. Red asterisks indicate the FSV strain sequenced in this study. Black circles at nodes represent support values of >0.70 from 1,000 bootstrap replicates. Additional information on the reference sequences used to build the tree is provided (Appendix Table 1). Scale bars indicate genetic distance. CVV, Cache Valley virus; FSV, Fort Sherman virus; L, large segment; M, medium segment; S, small segment.

Orthobunyavirus reassortment predominantly involves the M segment that encodes proteins responsible for viral receptor binding, thus potentially altering viral host range (*7*). Because CVV has been detected in several mosquito species (*6*), the human-derived FSV containing a CVV-like M protein might have a relatively broad host range, potentially including mosquito species that enable urban transmission cycles. This possibility is worrying because the Lambayeque region is a hot spot for the *Aedes* spp. mosquito–borne DENV, and during the COVID-19 pandemic, vector control activities were stopped (*8*). Although lack of another FSV-positive patient with febrile disease during 2020 in Lambayeque refuted an FSV outbreak, future outbreaks in humans cannot be excluded. Genetic monitoring of FSV will be required given that even single amino acid exchanges might affect the arboviral host range, as was demonstrated by the E1-A226V exchange in the Chikungunya virus envelope coding sequence that dramatically enhanced infection of *Aedes albopictus* (*9*).

The lack of studies describing FSV in humans is intriguing. One explanation could be the insufficient diagnostic capacity in areas where FSV potentially circulates. Another reason could be that human FSV infections are rare, potentially because of strong purifying selection that hinders the virus’s adaptation to human hosts (*10*). Our data highlight infection of humans with FSV in 2 ecologically distinct settings (coastal desert in Peru and coastal forest in Panama; https://www.oneearth.org) ≈2,000 km and 35 years apart in Latin America. The transmission cycle of both the human- and horse-derived FSV strains needs to be elucidated to identify risk groups and design intervention strategies. FSV should be considered in the differential diagnosis of febrile disease in Latin America, ideally including the development of robust serologic tests.

Appendix Additional information about Fort Sherman virus infection in human, Peru, 2020
