# Supplementary material for: Fort Sherman Virus Infection in Human, Peru, 2020
Source: Emerg Infect Dis. 2024 Oct;30(10):2211–4. doi: 10.3201/eid3010.240124 (PMC11431901; doi:10.3201/eid3010.240124)
Supplement: Appendix — Additional information about Fort Sherman virus infection in human, Peru, 2020 [file 24-0124-Techapp-s1.pdf]

Article DOI: https://doi.org/10.3201/eid3010.240124

EID cannot ensure accessibility for supplementary materials supplied by authors. Readers who have difficulty accessing supplementary content should contact the authors for assistance.

Fort Sherman Virus Infection in Human, Peru, 2020

Appendix

Materials and methods

Sampling

Serum samples were collected for the SARS-CoV-2 seroprevalence Study in Peru in collaboration with the Ministry of Health. Procedures were performed according to the approval of the Institutional Bioethics Committee VÍA LIBRE under protocol number 6528.

Molecular detection

For the screening testing for orthobunyavirus, a previously published broad reactive RT-PCR was used (*1*). The amplicon of the positive sample was sent for Sanger sequencing and then analyzed by BLASTN for virus identification. The FSV positive serum was then quantified by a real-time RT-PCR based on the viral polymerase gene using the primers FSHV-rtF (5′-TGTTGGTGATTGTGCATATATTGG), FSHV-rtR (5′-GGCGGACAACCATGTTTAATACT) and the probe FSHV-rtP (5′-ATCTAGCCAGTAGGTTATCTGCCACGCAGC), as previously published (*2*).

Sera were screened for the presence of dengue virus (DENV) using a commercial real-time RT-PCR (TIB Molbiol). Additional screening for other flaviviruses and alphaviruses was performed by broadly reactive nested RT-PCR, also using previously published tests (*3*,*4*).

Viral Isolation attempts

For virus isolation, monolayers of 1.6×10^5 cells per well were seeded in 12 well plates. The RT-PCR-positive serum was diluted at 1:10 and 1:100, inoculated into Vero E6, Vero FM, BHK and C6/36 cell lines, and incubated for 1 hour, as described previously (*2*). Cells were observed daily for 7 days for the presence of a cytopathic effect (CPE). After 7

days, cells were passaged, monitored daily and tested by real-time RT-PCR. Blind passages were repeated two times.

Evolutionary analysis

Maximum likelihood (ML) phylogenetic trees were performed using translated amino acid sequences of all three coding sequences using MEGA X (*5*) and 1,000 bootstrap replicates, a WAG amino acid substitution model, and complete deletion of all positions containing gaps in the alignment. Amino acid and nucleotide pairwise sequence distances were calculated in MEGA X using a pairwise deletion of all positions containing gaps in the alignment. Additional information on the selected orthobunyaviruses reference sequences used to build phylogenetic trees and for the sequence distance comparisons is detailed in Appendix Table 1.

Sequencing

Primers for each genomic segment are available in Appendix Table 2.

References

1. Lambert AJ, Lanciotti RS. Consensus amplification and novel multiplex sequencing method for S segment species identification of 47 viruses of the Orthobunyavirus, Phlebovirus, and Nairovirus genera of the family Bunyaviridae. J Clin Microbiol. 2009;47:2398–404. PubMed https://doi.org/10.1128/JCM.00182-09

2. de Oliveira Filho EF, Carneiro IO, Ribas JRL, Fischer C, Marklewitz M, Junglen S, et al. Identification of animal hosts of Fort Sherman virus, a New World zoonotic orthobunyavirus. Transbound Emerg Dis. 2020;67:1433–41. PubMed https://doi.org/10.1111/tbed.13499

3. Moureau G, Temmam S, Gonzalez JP, Charrel RN, Grard G, de Lamballerie X. A real-time RT-PCR method for the universal detection and identification of flaviviruses. Vector Borne Zoonotic Dis. 2007;7:467–77. PubMed https://doi.org/10.1089/vbz.2007.0206

4. Grywna K, Kupfer B, Panning M, Drexler JF, Emmerich P, Drosten C, et al. Detection of all species of the genus Alphavirus by reverse transcription-PCR with diagnostic sensitivity. J Clin Microbiol. 2010;48:3386–7. PubMed https://doi.org/10.1128/JCM.00317-10

5. Kumar S, Stecher G, Li M, Knyaz C, Tamura K. MEGA X: Molecular Evolutionary Genetics Analysis across computing platforms. Mol Biol Evol. 2018;35:1547–9. PubMed https://doi.org/10.1093/molbev/msy096

Appendix Table 1. Reference sequences used for evolutionary inferences*

Virus	Strain	L	M	S	Host detected	Country	Year†
Anadyr	LEIV-13128Kam	KT313717.1	KT313718.1	KT313719.1	*Aedes* sp.	Russia	1986
Batai	NM/12	KJ187038.1	KJ187039.1	KJ187040.1	Cattle	People's Republic of China	2012
Bunyamwera		X14383.1	M11852.1	D00353.1			1991‡
CVV Colombia	CoAr 3363	KX100108.1	KX100107.1	KX100106.1	*Aedes scapularis*	Colombia	1964
CVV Mexico	61D240	MH484342.1	MH484343.1	MH484344.1	*Mansonia titillans*	Mexico	1961
CVV USA	R132738d	OL555724.1	OL555725.1	OL555726.1	*Homo sapiens*	USA	2021
FSV (1965)	CbaAr426	KX100111.1	KX100110.1	KX100109.1	*Ochlerotatus albifasciatus*	Argentina	1965
FSV (1982)	AG83–1746	KX100114.1	KX100113.1	KX100112.1	*Psorophora varinervis*	Argentina	1982
FSV Argentina	SFCrEq231	KP063892.1	KP063893.1	KP063894.1	Horse	Argentina	2013
FSV Brazil	Barreiras	MN379835.1	N379834.1	MN379833.1	Horse	Brazil	2018
FSV Panama	86MSP18	NC_043617.1	NC_043615.1	NC_043616.1	*Homo sapiens*	Panama	1985
FSV Peru					*Homo sapiens*	Peru	2020
Ilesha	R5964	KF234075.1	KF234074.1	KF234073.1	*Homo sapiens*		1968
Kairi	BeAr8226	KR260738.1	KR260739.1	KR260740.1	*Ochlerotatus scapularis*	Brazil	1957
Lokern	FMS 4332	MG828823.1	MG820264.1	MG696865.2	*Culex tarsalis*	USA	1962
Maguari	OBS 6657	ARI46638	ARI46637.1	ARI46635.1	*Homo sapiens*	Peru	1998
Main Drain	72V2567	MH484306.1	MH484307.1	MH484308.1	*Aedes vexans*	USA	1972
Northway	0234	AVX27622	ABV68911.1	AXM42966.1	*Aedes* sp.	USA	1971
Playas	75V5758	ARI46654	ARI46653.1	ARI46651.1	*Aedeomyia taeniorhynchus*	Ecuador	1975
Potosi	89–3380	AXP32050	AXP32051.1	AXP32052.1	*Aedes albopictus*	USA	1961
Tensaw	TSV-FL06	ACV95628	ACV95623.1	ACV95624.1	*Anopheles crucians*	USA	2006

*CVV, Cache Valley virus; FSV, Fort Sherman virus.
†Isolation or detection year.
‡The sequence release date was used when information was not available.

Appendix Table 2. Primers used for amplification of complete coding sequence of FSV genomic segments*

Oligo name	Oligo sequence	Orientation
S segment		
FSV S F1	CGAGCGGTAGTAGTGTACTC	forward
FSV S F2	AGTAGTGTACTCCACACTAATAAC	forward
FSV S R1	AGTAGTGTGCTCCACCTAAAACT	reverse
FSV S R3	GGATAGAATTTGAATGTTCCTAAGAA	reverse
FSV S R2	TTCAGATCCTGGGAAGAATGAG	reverse
FSV S F3	CTTTGGCTGAAAAAAATGGAATTACA	forward
FSV S R4	TTTCAGATCCTGGGAAGAATGAG	reverse
FSV S F4	CTTTGGCTGAAAAAAATGGAATTAC	reverse
	M segment	
FSV M F14	CAGATGTAAGACTTGGTTATGTAG	forward
FSV M F1	GTAGTAGTGTACTACCGATACAAT	forward
FSV M F2	CTTAGTCTAATGACAAAGACATACA	forward
FSV M F3	AGATCTTAGTCTAATGACAAAGACA	forward
FSV M F4	ATTGATGAAAAGGACAGCAGATGTA	forward
FSV M F5	GTTATCTACTGATGCCAATTTTCATG	forward
FSV M F6	AAATAACAAGCATACTAGCAAGAAGA	forward
FSV M F7	ACAAGCATACTAGCAAGAAGAAATG	forward
FSV M F8	TTAAGGCAATCAGAGCCCAGAAA	forward
FSV M F9	GGAGTCTGGAAATTTGGACAGT	forward
FSV M F10	GGAGTCTGGAAATTTGGACAGTA	forward
FSV M F11	GGCAGATAAAGGCGTTTGTACA	forward
FSV M F12	GAATCAGCATACATCTTGTCTAGTA	forward
FSV M F13	CCAGGCAATAATTACTGCACTGA	forward
FSV M R1	CCTCCTCTTTATCTATGGTTATAGT	reverse
FSV M R2	GCCTCCTCTTTATCTATGGTTATA	reverse
FSV M R3	CACTTTTTGCAGCTCTTGTTGTA	reverse
FSV M R4	GCACTTTTTGCAGCTCTTGTTGT	reverse
FSV M R5	TCACCGTTATACTTTATGCCTCTTT	reverse
FSV M R6	TCACCGTTATACTTTATGCCTCTT	reverse
FSV M R7	GGTTGAAACTCGTGTAATAATCACA	reverse
FSV M R8	GCTCAATTCAGTGTTATTTATGTATG	reverse
FSV M R9	CTCCTATTCTGGCTCAATTCAGT	reverse

Oligo name	Oligo sequence	Orientation
FSV M R10	GAAGTAGTACAATCCACTATCACAT	reverse
FSV M R11	GGAAATTGTCTTTGGTTTGGACAT	reverse
FSV M R12	GGAAATTGTCTTTGGTTTGGACATT	reverse
FSV M R13	CTGGAGCTCTATCTCATCTGTT	reverse
FSV M R14	ACTGGAGCTCTATCTCATCTGT	reverse
FSV M R15	TAGCTTTCAAAACACGATGGACAT	reverse
FSV M R16	GCAGGAGTAGCTTTCAAAACAC	reverse
FSV M R17	TGTGCTACCGATATCAAAAACAG	reverse
M5′Rev	CCT GTC GACAGTAGTGTGCT	reverse
	L Segment	
FSV L F01	TGTAGTAGTGTACTCCTATATAAAGA	forward
FSV L F02	TCCTGGATTATAAAGTGTCAGTTG	forward
FSV L F03	ATGGGATCTATGCCACCTAGAT	forward
FSV L F04	GTAGAACCTTCAACATGGACAGA	forward
FSV L F05	CAGAACCTTCAACATGGACAGAT	forward
FSV L F06	GCTTCTTGTCTCAGAATAATACACT	forward
FSV L F07	AGATGCCAGCGAATTGTCTCTT	forward
FSV L F08	AAATGATGAAAGGAATCTGGATTCTA	forward
FSV L F09	AAATGATGAAAGGAATCTGGATTCT	forward
FSV L F10	AGTATAGACTATCGAACCCATTGTT	forward
FSV L F11	AAGTATAGATTATCGAACCCATTGT	forward
FSV L F12	AGGAAAGGTGCAAGTTAAATACTGAT	forward
FSV L F13	GGAAAGGTGCAAGTTAAATACTGAT	forward
FSV L F14	CTACTGTCCAATGTGCTTGATCAA	forward
FSV L F15	CCTTTCAGAATGATCTAATACTAGA	forward
FSV L F16	TCTTACTACCAAGTGTTGGTGATT	forward
FSV L F17	CACAGTGCCAGTGGCTAACA	forward
FSV L F18	AACACAGTGCCAGTGGCTAACA	forward
FSV L F19	TGAGCTCACTAGTAGAATCAGATAT	forward
FSV L F20	CAAGCATTTGACAGGCACAGAA	forward
FSV L F21	TGAGGTGATAGATAACTTCCAGTAT	forward
FSV L F22	GTGCTTGACGAAAAATATAACTACTA	forward
FSV L F23	GTGCTTGACGAAAAATATAACTACTAT	forward
FSV L F24	AACACAAGAATTGCTGTCACTGAA	forward
FSV L F25	ACACAAGAATTGCTGTCACTGAAT	forward
FSV L F26	CTAGTGACAAATGAATGGTCTTCT	forward
FSV L R01	CCATAACAGGCAAAATTAATGAAGTA	reverse
FSV L R02	TCCATAACAGGCAAAATTAATGAAGT	reverse
FSV L R03	ACCTTTCAGCTGAGTAAGCACT	reverse
FSV L R04	GACTATTCCACCTTTCAGCTGA	reverse
FSV L R05	CATAGGTTTTCATATTGGCCAACAT	reverse
FSV L R06	CATATTGGCCAACATTACCATCTA	reverse
FSV L R07	CTTCAGATGCTTCCTTTATTTGTGA	reverse
FSV L R08	GTTTAGCACATCCATAAGGGATACT	reverse
FSV L R09	TTTAGCACATCCATAAGGGATACT	reverse
FSV L R10	TAGCATTGAAATAAAAGGGTAGATAGA	reverse
FSV L R11	AGCATTGAAATAAAAGGGTAGATAGA	reverse
FSV L R12	GCTGCCAAATCTTCATAATTACAGT	reverse
FSV L R13	CACATTATAACGTATTTCTTCTTCTG	reverse
FSV L R14	AGCACAACTATGGACATAACTAGA	reverse
FSV L R15	ATACTTTGCTGCGTGGCAGAT	reverse
FSV L R16	ATACTTTGCTGCGTGGCAGATA	reverse
FSV L R17	CTGTGTCTAGAGTTAAGTATCTAAGT	reverse
FSV L R18	TCTGCACAAACCTCTGTGTCTA	reverse
FSV L R19	CTGATAAGCCTGCGGGAAAGT	reverse
FSV L R20	TACTTTGACCTTGTGTTCTGTTGA	reverse
FSV L R21	ACTTTGACCTTGTGTTCTGTTGAT	reverse
FSV L R22	CTATTTAAATCTGCCTGGGTTAGA	reverse
FSV L R23	TCTGGGTTAGAGCTTGCATTCT	reverse
FSV L R24	ATGTTCGGTTGTAGAAACCTGATA	reverse
FSV L R25	TATGTTCGGTTGTAGAAACCTGAT	reverse
FSV L R26	GTGTCCTCATCACCATTACATGA	reverse
FSV L R27	GCTGATGGCAAAGAGAACATGT	reverse
FSV L R28	GTGTGCTCCTATATAAAGAAATTGAT	reverse
FSV L R29	TGAGTAGTGTGCTCCTATATAAAGA	reverse

*FSV, Fort Sherman virus.

Appendix Table 3. Monthly detection ration of Dengue virus in department of Lambayeque, Peru, 2020.

Month	Positives	Total	Frequency
January	4	14	28.6%
February	106	154	68.8%
March	120	194	61.9%
April	114	122	93.4%
May	24	31	77.4%
June	4	6	66.7%
July	18	27	66.7%
August	3	7	42.9%
September	2	2	100.0%
October	5	6	83.3%
November	4	14	28.6%
December	6	6	100.0%
Total	410	583	70.3%

Appendix Table 4. Evolutionary divergence between Fort Sherman virus from Peru and related orthobunyaviruses*

Genomic segments	Human-derived FSV		Horse-derived FSV		CVV		Potosi		Tensaw		Maguari		Northway		Playas	
	N	A	N	A	N	A	N	A	N	A	N	A	N	A	N	A
L	2.0	0.8	5.0	1.9	23.4	12.4	23.7	12.5	21.7	9.4	21.9	10.0	21.2	10.3	21.9	10.4
M	2.3	1.5	24.6	17.3	9.0	3.9	38.3	44.0	26.7	20.6	24.7	14.9	24.3	15.7	25.1	14.6
S	1.0	0.0	3.0	0.0	12.8	3.9	11.8	4.3	11.4	3.4	10.1	4.3	11.6	1.7	15.5	4.3

*Evolutionary analyses were performed using MEGA10 (Appendix, https://wwwnc.cdc.gov/EID/article/30/10/24-0124-App1.pdf). N, indicates percent nucleotide sequence divergence of coding sequence; A indicates percent divergence of translated amino acid sequences. CVV, Cache Valley virus; FSV, Fort Sherman virus.

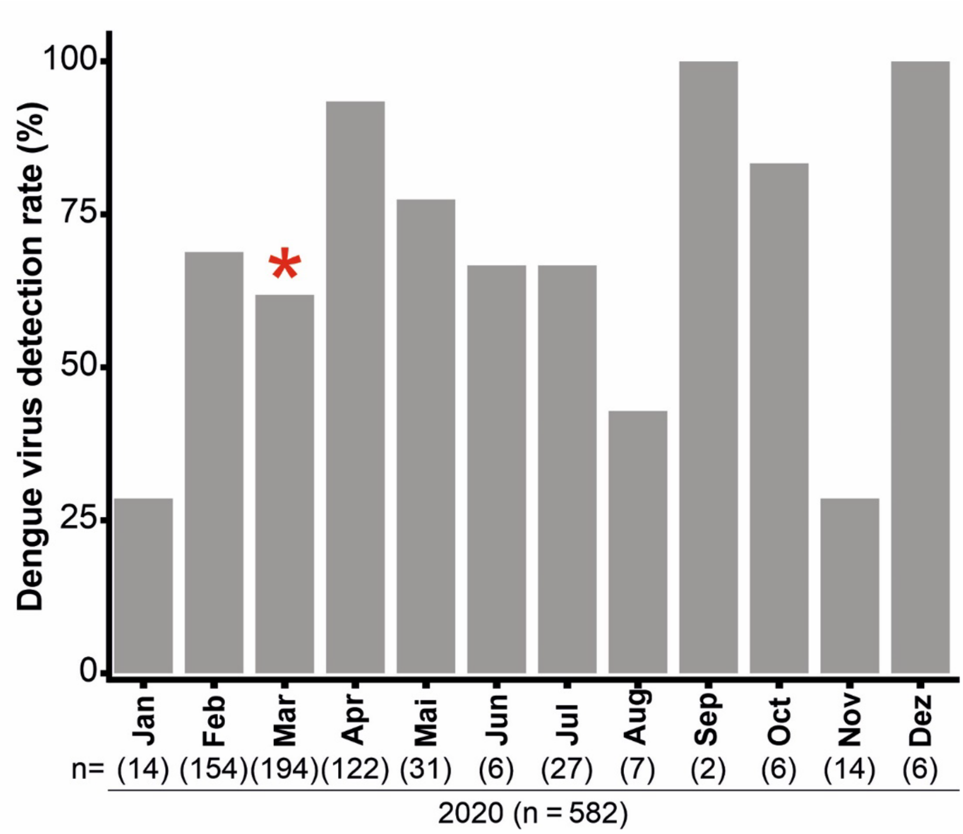

Appendix Figure. Detection rate of dengue virus by real-time RT-PCR among febrile patients in the Lambayeque department, Peru, in 2020. The red asterisk indicates the month a patient tested positive for FSV. The total number of sera tested per month is given in brackets.
